# As Signals From the Kawasaki-Like Illness During the COVID-19 Pandemic: Is It Possible That the Incidence of IgA Nephropathy May Increase in the Future

**DOI:** 10.3389/fmed.2021.737692

**Published:** 2021-11-02

**Authors:** Yasin Abdi Saed, Weiwei Xu, Hasnaa Yaigoub, Hasna Tirichen, Lili Guo, Li Cheng, Yafeng Li

**Affiliations:** ^1^Department of Nephrology, Graduate School of Shanxi Medical University, Taiyuan, China; ^2^Institutes of Biomedical Sciences, Shanxi University, Taiyuan, China; ^3^Shanxi Provincial Key Laboratory of Kidney Disease, Taiyuan, China; ^4^Department of Nephrology, The Fifth Hospital (Shanxi Provincial People's Hospital), Taiyuan, China

**Keywords:** SARS-CoV-2, COVID-19, mucosal immune, Kawasaki-like disease, IgA nephropathy

## Introduction

Coronavirus disease (COVID-19), caused by a novel beta coronavirus, severe acute respiratory syndrome coronavirus 2 (SARS-CoV-2), has been dominating our lives for over a year now, affecting every aspect from health, economy to social interactions. In addition to known complications of viral infections such as heightened immune responses, COVID-19 presents with serious multiorgan sequelae that need to be urgently addressed. Lucio Verdoni et al. reported that the SARS-CoV-2 epidemic is associated with a high incidence of a severe form of Kawasaki-like disease in Bergamo province in Italy on Lancet which draw attention to the complications of COVID-19 (1).

The etiology of Kawasaki disease is currently not fully understood. Direct viral infections, superantigen reactions and autoimmunity are thought to be linked to its onset. Magali Noval Rivas and colleagues observed that in the Kawasaki disease vasculitis mouse model, the intestinal barrier was damaged and, secretory immunoglobulin A (sIgA) secretion was increased. The damaged intestinal barrier caused sIgA leakage and sIgA-C3 complex in vascular tissue and glomeruli deposit, and thus promoting the occurrence of arteriovasculitis and abdominal aorta dilation (2). Patients with acute-phase Kawasaki disease have increased serum sIgA concentration and signs of intestinal barrier damaged. Intravenous immunoglobulin (IVIG) treatment can reduce the permeability of the intestinal barrier and serum sIgA concentration while reducing IgA deposition in vascular tissue (3). These evidence indicate that sIgA and intestinal barrier permeability play an important role in the occurrence and development of Kawasaki disease. The mucosal barrier immunity and mucosal barrier damage caused by SARS-COV-2 may be the cause of Kawasaki-like disease outbreak during the epidemic.

While IgM and IgG isotypes have received the most attention in the study of respiratory infection, mucosal and systemic IgA responses, which may play a crucial role in disease pathogenesis, have gotten far less consideration. knowing that viremia is a frequent complication of SARS. SARS-CoV-2 would be anticipated to produce secretory IgA (sIgA) and induce strong mucosal immunity. As well, IgA-mediated interactions with pathogenic microbes have been demonstrated to contribute to mucosal antiviral defense by preventing pathogens from adhering to the cell surface (4). Further, so recent research has discovered that sIgA can stimulate the synthesis of interleukin (IL)-6, IL-8, monocyte chemoattractant protein-1, and granulocyte-macrophage colony-stimulating factor throughout human lung fibroblasts (5). It's also been suggested that sIgA and IgG work together to promote antibody-dependent cellular cytotoxicity (ADCC) (6). The role of serum IgA, in contrast to mucosal IgA, is mostly unknown. Previous research has revealed that IgA mediates either pro- or anti-inflammatory actions in innate immune cells, indicating that IgA may play a role in autoimmune disorders and immunological hyperactivation regulation (7). In a number of myeloid cells, monomeric binding of serum IgA to the Fc alpha receptor (FcRI) has been hypothesized to mediate inhibitory activity via receptor inhibitory signals (8). In contrast, IgA and pathogen crosslinking of FcRI allows activating signals to be sent, resulting in phagocytosis, respiratory burst, ADCC, increased antigen presentation, degranulation, and cytokine release (9).

Antibody isotype switching can be induced by cytokines such as transforming growth factor (TGF)- and interleukin-10 (10). Also increased levels of TGF- and IL-10, which drive antibody switching in SARS-CoV-2 infection, might explain the increased IgA production. Considering the roles of mucosal and systemic IgA in COVID-19, stimulating IgA synthesis (by activating canonical TGF-signaling with lactoferrin) (11). It's also worth mentioning that a new treatment for severe COVID-19 has been proposed using retinoic acid to increase lactoferrin-induced IgA responses (12).

IgA antibodies in the mucosa are polyreactive and have a low affinity for bacterial antigens. Mucosal pathogens and vaccines can cause high-affinity and T-cell-dependent IgA responses (13). SARS-CoV-2 can cause strong mucosal immunity to induce sIgA production, and the serum SARS-CoV-2-specific IgA level was found to have a significant positive association with the APACHE-II score of critically ill patients with COVID-19 (14). The production of large amounts of sIgA is an important step in the pathogenesis of Kawasaki disease.

Kawasaki disease patients with digestive tract symptoms are more likely to develop IVIG resistance and coronary artery lesions.SARS-CoV-2, generally, first attacks the respiratory system and causes serious infections. 61.3% of the 318 SARS-CoV-2-infected patients from nine hospitals in the United States reported at least one gastrointestinal symptom, the most common gastrointestinal symptoms were anorexia (34.8%), diarrhea (33.7%) and nausea (26.4 %) (15). In an *in vitro* organoid model, SARS-CoV-2 can effectively infect human small intestine organoids, and replicate. Digestive endoscopy sampling showed that in the patient's stomach, duodenum and rectum, the epithelial cells expressed the viral host receptor ACE2, and the viral nucleocapsid protein was detected in the cytoplasm, and a large amount of pulp infiltrating was visible in the lamina propria Cell, lymphocyte and interstitial edema (16). The SARS-CoV-2 can directly infect the respiratory system and digestive system causing mucosal barrier damage, which can be regarded as a high-risk factor for Kawasaki-like disease.

To sum up, we propose the hypothesis that SARS-CoV-2 invades the mucosa of the respiratory tract and digestive tract, causing damage to the mucosal barrier and increases secretion of sIgA, sIgA leaks into the blood and promotes the deposition of IgA-C3 complex in the cardiovascular lesions to cause Kawasaki-like disease.

## Discussion

IgA nephropathy (IgAN) is considered as the most common primary glomerulonephritis globally. The pathological feature of IgAN is the deposition of IgA in the mesangial area of the glomeruli; however, its pathogenesis is unclear. IgAN is a multifactorial disease. Recent studies have shown that respiratory and intestinal mucosal immunity is closely related to the pathogenesis of IgAN. Some IgAN patients have prodromal symptoms such as upper respiratory tract (tonsillitis, pharyngitis) and digestive tract infections within hours or days before the onset of illness. IgAN patients are more likely to have gastrointestinal symptoms such as celiac disease. Pathological changes similar to human Kawasaki-like disease and IgA Nephropathy(IgAN) were observed in the damaged intestinal mucosal barrier of the Kawasaki disease mice model.

Moreover, the spike protein of SARS-CoV-2 binds to ACE2 receptors on the surface of targeted cells (17). ACE2 is widely found in various tissues, particularly in the proximal tubules' apical brush borders and to a lesser extent in kidney podocytes (18). The link between ACE2 and COVID-19 has piqued curiosity as a result of this discovery. The presence of viral components (e.g., spike protein) in renal tissue and virus-like particles within epithelial cells was confirmed by histological results from postmortem tissues (19). Furthermore, Pan et al. assert that the kidney is predisposed to COVID-19 because of ACE2 expression (20). In the light of the Kawasaki-like disease outbreak during the COVID-19 pandemic, we hypothesized that IgAN may be another possible complication of COVID-19.

**Figure 1:**
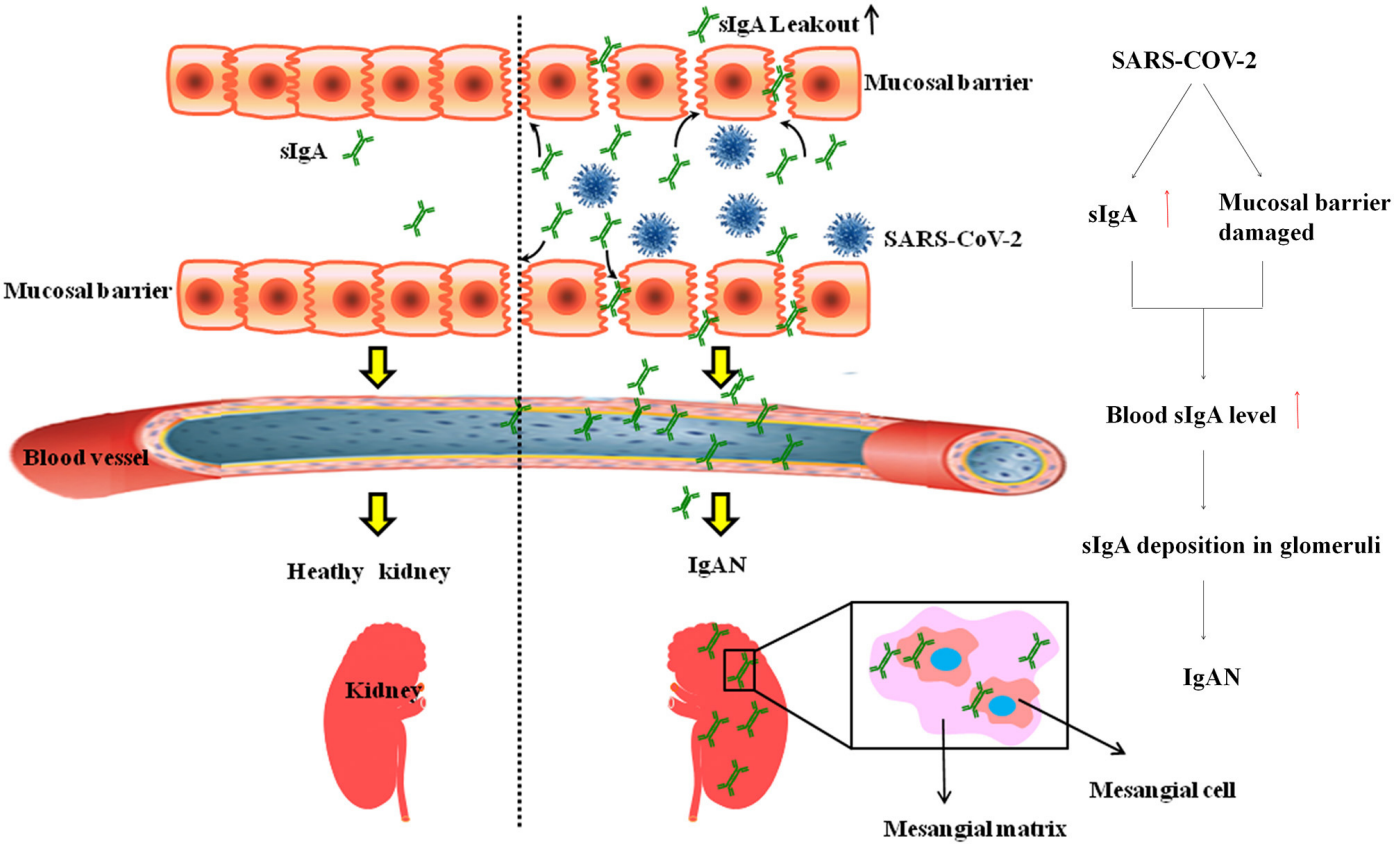
SARS-COV-2 Lead to IgAN. SARS-CoV-2 invades the mucosa of the respiratory tract and digestive tract, increased gut permeability leads to leakage of sIgA and promotes the deposition of IgA-C3 complex in the mesangial area of the glomeruli to cause IgAN.

We collected urine from 864 patients with COVID-19 from Hubei Provincial People's Hospital for routine urine testing and found that 233 (30%) patients had urinary occult blood. Hematuria is the most common clinical manifestation of IgAN (21). The onset of IgAN is insidious and often manifests as asymptomatic hematuria. After the onset of gross hematuria, urinary erythrocytes can disappear or can be converted to microscopic hematuria. Some patients with IgAN often have paroxysmal gross hematuria associated with upper respiratory tract infections. Therefore, we speculate that some COVID-19 patients who presented with occult blood, this latter is caused by IgAN complications. We propose the hypothesis that SARS-CoV-2 invades the mucosa of the respiratory tract and digestive tract, causing damage to the mucosal barrier and increases secretion of sIgA, sIgA leaks into the blood and promotes the deposition of IgA-C3 complex in the mesangial area of the glomeruli to cause IgAN (Figure 1).

When patients with COVID-19 have hematuria, we first consider the acute kidney injury caused by SARS-COV-2 and ignore IgAN. The onset of IgAN is hidden, and the diagnosis often depends on renal puncture. During the epidemic, our focus is mainly on whether the patient's nucleic acid test turns negative and whether the symptoms of pneumonia are alleviated, and kidney pathological examinations are often ignored. In particular, the lack of experienced pathologists in developing countries is more likely to ignore IgAN diagnosis. Most patients undergo renal pathology only when they find abnormal renal function during the medical examination. Therefore, we suspect that the incidence of IgAN may increase in the future.

Lately, there is an important question concerning the renal risks of vaccination against SARS-CoV-2. With the advent of mRNA-based vaccinations, concerns about the possibility of renal adverse effects have arisen. Flare-ups of nephrotic syndrome associated with minimal glomerular damage or episodes of hematuria have recently been reported in patients with IgA-deposed nephropathy following vaccination. Based on the data reported, it is currently impossible to conclude that there is a causal link. To our knowledge, nine cases of hematuria due to IgA deposit nephropathy have been reported so far (22–25).

Whilst the correlation does not necessarily imply the cause, symptoms timing should be seen as the inciting event shortly after the vaccine, The development of anti-glycan antibodies that cross-react with pre-existing under-galactosylated IgA1 is one proposed explanation for IgAN. Furthermore, an mRNA-based vaccine may stimulate higher T follicular helper and subsequent B-cell responses in the germinal center, which potentially resulting in more robust antibody production. Given elevated IgA level, another possibility is an increase in pathogenic IgA production, similar to the influenza vaccination. A recent preprint study also indicate that healthy people who received mRNA vaccinations had strong spike-specific IgA responses (26).

In conclusion, we hypothesized that IgAN may be another serious complication of COVID-19 as well COVID-19 mRNA vaccine and the incidence of IgAN may increase in the future. IgAN has a long course and poor prognosis. Early diagnosis and intervention are of great significance for improving the prognosis and quality of life of patients with COVID-19.

## Author Contributions

YA wrote original draft. WX, HY, HT, and LG did the review and editing. YL dealt with the project administration and supervision. All authors contributed to the article and approved the submitted version.

## Funding

This work was supported by the National Natural Science Foundation of China (No. 82170716) and the COVID-19 Project Fund of Shanxi Province Health Commission (No. 15).

## Conflict of Interest

The authors declare that the research was conducted in the absence of any commercial or financial relationships that could be construed as a potential conflict of interest.

## Publisher's Note

All claims expressed in this article are solely those of the authors and do not necessarily represent those of their affiliated organizations, or those of the publisher, the editors and the reviewers. Any product that may be evaluated in this article, or claim that may be made by its manufacturer, is not guaranteed or endorsed by the publisher.
